# Optimization of CCGT power plant and performance analysis using MATLAB/Simulink with actual operational data

**DOI:** 10.1186/2193-1801-3-275

**Published:** 2014-06-01

**Authors:** Naimul Hasan, Jitendra Nath Rai, Bharat Bhushan Arora

**Affiliations:** Department of Electrical Engineering, Jamia Millia Islamia, New Delhi, India; Department of Electrical Engineering, Delhi Technological University, Delhi, India; Department of Mechanical Engineering, Delhi Technological University, Delhi, India

**Keywords:** Combined cycle, Optimization, Model, Gas turbine, Efficiency

## Abstract

In the Modern scenario, the naturally available resources for power generation are being depleted at an alarming rate; *firstly* due to wastage of power at consumer end, *secondly* due to inefficiency of various power system components. A Combined Cycle Gas Turbine (CCGT) integrates two cycles- Brayton cycle (Gas Turbine) and Rankine cycle (Steam Turbine) with the objective of increasing overall plant efficiency. This is accomplished by utilising the exhaust of Gas Turbine through a waste-heat recovery boiler to run a Steam Turbine. The efficiency of a gas turbine which ranges from 28% to 33% can hence be raised to about 60% by recovering some of the low grade thermal energy from the exhaust gas for steam turbine process. This paper is a study for the modelling of CCGT and comparing it with actual operational data. The performance model for CCGT plant was developed in MATLAB/*Simulink*.

## Introduction

With the advent of technological advancement, the dependency of human race on electricity has increased manifolds and keeping in mind the uncontrollable power requirement in almost every minute human activity methods are being taken up to exploit the present natural resources like coal, solar etc. Moreover, necessary up-gradation can be done so as to generate more power than the plant used to do in its normal running time.

A CCGT is one such advancement in the field of power generation. It consists of two units (a) the steam turbine unit and (b) the gas turbine unit. The net power output is the summation of both the independent units.

The two units while being physically independent, depend on each other for their operation. The gas turbine unit is fired first. This results in hot exhaust gases from the turbine. This hot exhaust gas is used to operate the boiler of the steam turbine generating steam. Once steam is generated the operation of the steam turbine starts.

As the above explanation shows, the steam turbine operates from the energy wasted at the exhaust of the gas turbine. Consequently no separate fuel or energy is required to operate the steam turbine. This results in considerable saving of energy while increasing the power generated.

Conversion of hot gases from the exhaust of the gas turbine to heat required for the boiler is done by the Heat Recovery Steam Generator (HRSG) unit.

The input temperature to a steam turbine is about 540°C and the exhaust can be maintained at the atmospheric pressure, due to design consideration the input temperature is limited and the efficiency of the about 40%. The input temperature of the gas turbine can be as high as 1100°C but the exhaust temperature can be lowered to about 500-600°C, the efficiency of a gas turbine is about 33%. It can be seen that to obtain higher efficiencies the exhaust of the gas turbine can used to drive the steam turbine giving efficiency up to 60% (Black & Veatch 1996).

The plant consists of a compressor, combustor, gas turbine, waste heat recovery boiler, steam turbine, and generator(s).

**Figure 1:**
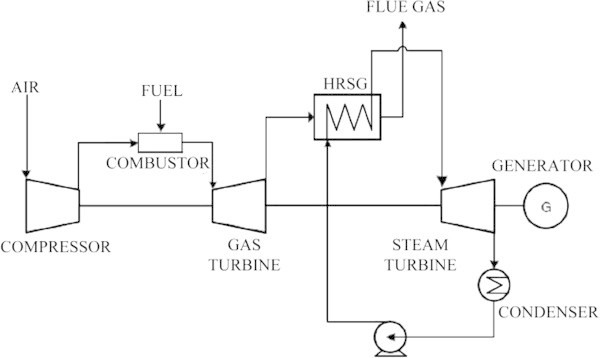
**Combined cycle gas turbine.**

The air is provided in the compressor which compresses the air and passes it to the combustion chamber, where the compressed air is mixed with the fuel and burnt. The mixture is then sent to the gas turbine where it expands and rotates the turbine (Figure 1). The heat of the flue gas is recovered in HRSG (Heat Recovery Steam Generator) which is used to supply steam to the steam turbine at proper temperature and pressure. Plant power output is the sum of the gas turbine and the steam turbine outputs (Horlock 2003; Kehlhofer et al. 2009; Drbal Lawrence et al. 1996; Lalor & O'Malley 2003).

## CCGT thermodynamics

The airflow (W) in the gas turbine is given as1

Where Ti is ambient temperature and P_a_ denotes the atmospheric pressure. *W*_*a*_ is air flow with the assumption that *P*_*a*_ = *P*_*a*0_.

The compressor discharge temperature is given as23

*P*_*ro*_ is the design compressor pressure ratio and y is the ratio of specific heats.

The gas turbine inlet temperature T_f_ (K) is given by (Kakimoto & Baba 2003)4

Where W_f_ is fuel flow per unit its rated value, ‘o’ denotes rated value, W denotes the airflow and T_d_ denotes the compressor discharge temperature.

Gas Turbine exhaust temperature T_e_ (K) is given by (Kakimoto & Baba 2003)5

Where *η*_*t*_ is turbine efficiency. The exhaust gas flow is practically equal to the airflow.

The efficiency of a combined cycle (unfired) is given as, Horlock (Horlock 2003)6

Where *η*_*cc*_is the efficiency of the combined cycle, *η*_*gt*_ is the efficiency of Gas Turbine and *η*_*st*_ is the efficiency of Steam Turbine. The thermal efficiency of the simple gas turbine cycle is given as (Al-Zubaidy & Bhinder 1996)7

Where, *a* = *η*_*c*_*η*_*t*_*k*_1_.

Where *p*_*p*_ is the isentropic temperature ratio (T_2_/T_1_), k_1_ is the cycle maximum temperature ratio (T_3_/T_1_).

Differentiating (6) gives (Kehlhofer et al. 2009)8

The overall efficiency improves with the increase in gas turbine efficiency if9

From equation (8) and (9) one obtains:10

The above calculations were done using the following parameters: Pressure Ratio: 8 to 16; Air Fuel Ratio: 50 to 65; Plant Rating: 122 MW for Steam Turbine and 104 MW for Gas turbine.

## Description of simulink model

**Figure 2:**
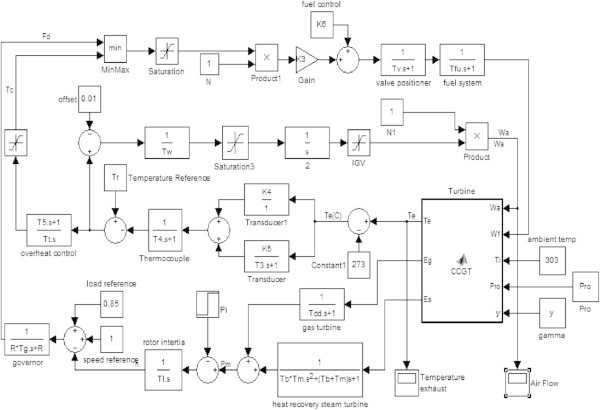
**Simulink model of combined cycle gas turbine.**

**Figure 3:**
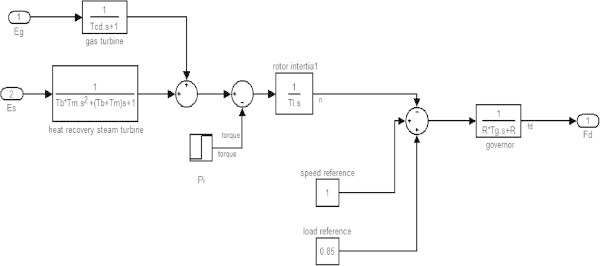
**Speed/Load control block.**

**Figure 4:**
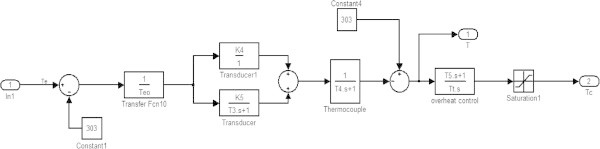
**Temperature control block.**

**Figure 5:**
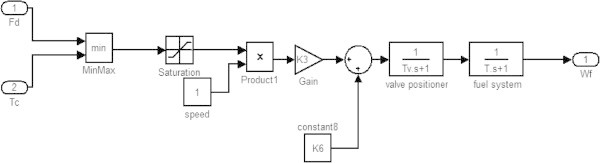
**Fuel control block.**

**Figure 6:**
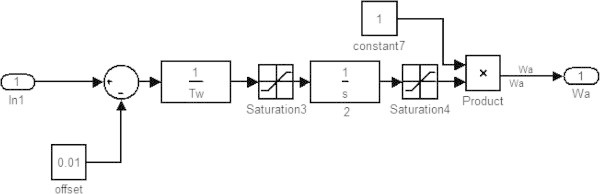
**Air control block.**

Several CCGT models have been developed in past few decades with to describe the behavior of gas turbine. The basic gas turbine model equations (Rowen Model II) (Rowen 1983; de Mello & Ahner 1994) for a single shaft system were given by Rowen in 1992. Figures 2, 3, 4, 5 and 6 shows a dynamic model of a combined cycle gas turbine.

This model consists of various blocks describing various parameters whose variations have to be studied in order to optimize the performance of combined cycle. There are blocks related with speed/load, temperature control, fuel control, air control and other blocks for gas turbine, waste heat recovery boiler/steam turbine, rotor shaft, and temperature transducer.

### Speed/Load control block

**Figure 7:**
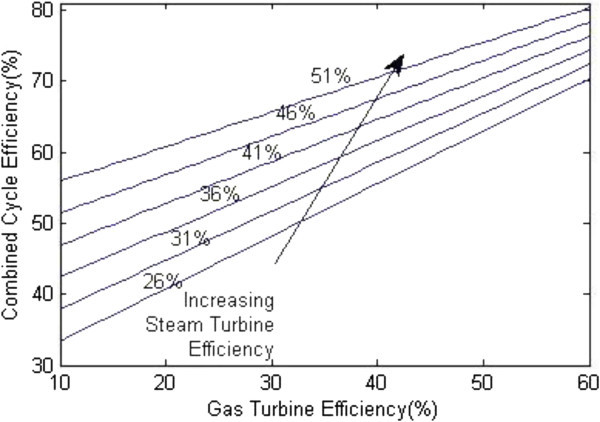
**Combined cycle efficiency versus Gas turbine efficiency.**

The speed/load control block (Kunitomi et al. 2001) is used to determine the fuel demand F_d_ in accordance with a reference load reference and rotor speed reference (1-n). The value of n is determined with the help of blocks representing the net energy supplied to the gas turbine E_g_ andthe energy collected by the heat recovery boiler and steam turbine E_s_. The output of these blocks is the power P_g_ and P_s_ respectively which when summed up gives the plant power output P_m_. The rotor speed varies if there is any difference between power output P_m_ and load power P_l_ represented by the reference block of torque. After the value of n is obtained it is compared with the speed reference block and load reference blocks whose output when applied to governor gives the fuel demand F_d._ (Figure 7).

### Temperature control block

**Figure 8:**
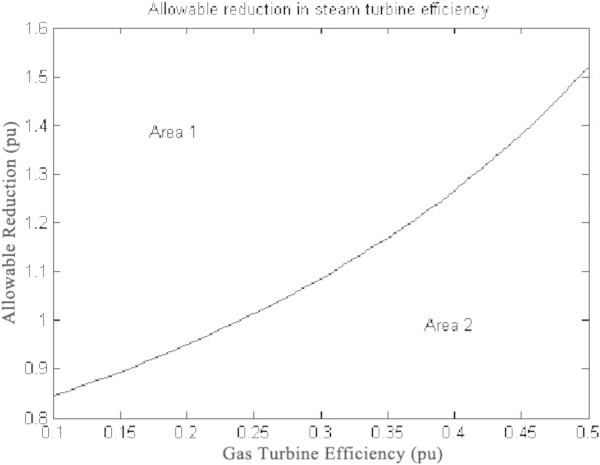
**Gas Turbine efficiency versus rate of change of steam turbine efficiency.**

The temperature control block (overheat control) is for controlling the exhaust temperature (T_e_^0^C) of the gas turbine so that the gas turbine does not get injured. The temperature is measured with the help of various transducers and thermocouple as shown in block diagram. The output of the thermocouple (electrical signals) is compared with a reference value (constant 4) (Rai et al. 2013a). According to the difference in values of thermocouple output and reference value the temperature control (Overheat control and Saturation 1) produces temperature control signal T_c_. Then the output of the temperature control is combined with speed/load control to determine the fuel demand (using low select value) (Figure 8).

### Fuel control block

The fuel control block (valve positioner and fuel control) performs according to the minimum value provided by the speed/load control and temperature control and determines the fuel flow W_f_. The Min-Max block selects the minimum value between speed/load control and temperature control and the saturation controls the maximum value of the fuel flow. The output of the saturation is modified by various control blocks and then is input to the valve positioner block which controls the positioning of the valve for the fuel flow. The output of the valve positioner is then input a fuel system control block which gives the value of the fuel flow W_f_.

### Air control block

The air control block (saturation 3 and saturation 4) is to adjust the air flow in the gas turbine to attain a desired exhaust temperature so that the temperature is kept below a reference temperature by an appropriate offset. This arrangement is used to control the compressor inlet guide vanes (IGV). The exhaust temperature of gas turbine T_e_ is compared with a reference temperature which gives the value in ln1. The value of ln1 is compared with offset block and then the output is sent to the air control blocks. The air control blocks adjust the opening of IGV according to the exhaust temperature of the gas turbine. The air flow in gas turbine is used to control the exhaust temperature in the gas turbine.

## Results and discussions

**Figure 9:**
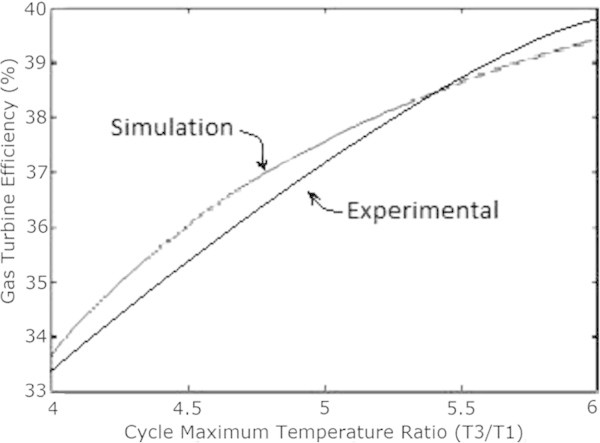
**Gas turbine efficiency versus Maximum cycle temperature ratio.**

**Figure 10:**
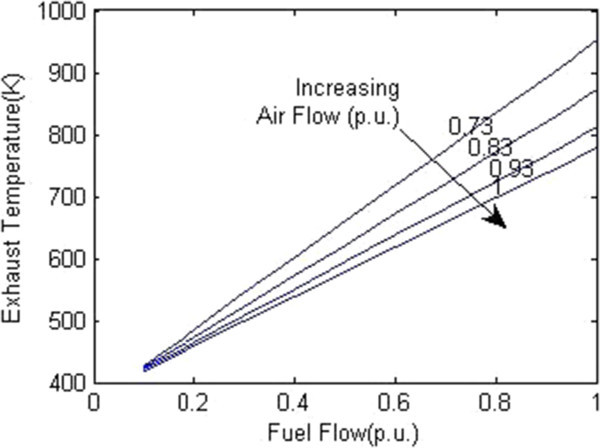
**Exhaust temperature versus Fuel flow.**

**Figure 11:**
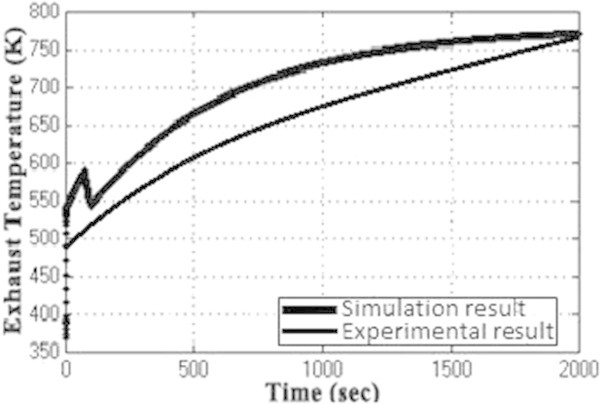
**Exhaust temperature (K) versus Time.**

**Figure 12:**
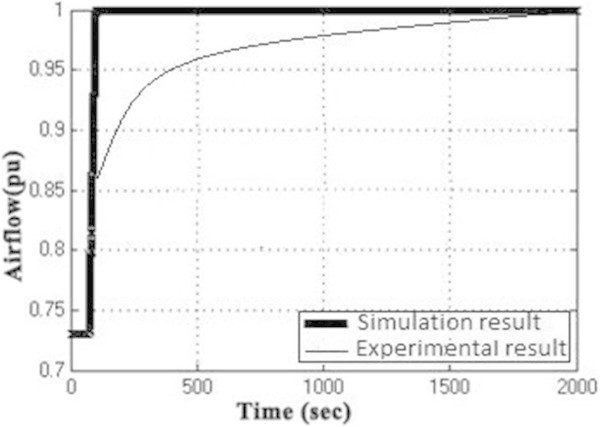
**Air flow (p.u.) versus Time.**

Figure 7 shows the plot between overall efficiency and gas turbine efficiency with varying steam turbine efficiency as per the equation (6) (Rai et al. 2013b). It can be seen that combined cycle efficiency increases with the increase of both gas turbine efficiency and the steam turbine efficiency.The graph in Figure 8 shows the change in gas turbine efficiency for various rate of change in steam turbine with gas turbine efficiency keeping steam turbine efficiency constant = 24%. It shows that if the efficiency of steam turbine is kept constant and the efficiency of gas turbine is varied, the overall efficiency of the combined cycle can be increased but if the value of derivative falls below the R.H.S. the efficiency of combined cycle would drop. The area I shows the region in which there is allowable reduction in steam turbine efficiency with respect to the gas turbine efficiency.Figure 9 shows the variation of gas turbine efficiency with cycle maximum temperature ratio (T3/T1) keeping the pressure ratio constant in accordance to equation (7). It shows that the turbine efficiency can be increased by increasing the maximum temperature ratio of Brayton cycle. The optimal value of T3/T1 observed as 5.5 This matches with the safe limit of operation of the plant beyond which the plant starts overheating.The plot (Figure 10) shows how exhaust temperature of the gas turbine varies with the fuel flow as per equation (5). It can be seen that the exhaust temperature increases with the increase in fuel flow. As more energy is supplied temperature increases.Figure 11 shows the variation of exhaust temperature with time. The exhaust temperature first increases due to more fuel flow till a reference temperature where it is controlled by air flow so that it does not rise any further as it would damage the turbine. From Figure 12 it can be seen that the IGV (Inlet Guide Vanes) start opening to allow more flow of air and thus reducing the exhaust temperature as can be seen by the drop in the exhaust temperature.The difference in the plots (Figure 11 and Figure 12) of the experimental graph and the simulation is explained by the following:

- Simulation is based on numerical analysis which results in prediction errors while experimental result is the what is actually obtained
- Simulation is based on parameters which do not get affected during the simulation process while the parameters provided during experiment can change due to factors beyond the control of the researcher.

The results on reports were generated based on the following plant ratings and the parameters provided in Table 1 along with gas turbine model described in Appendix.

**Table 1:** **System parameters**

Symbol	Description	Value
*T*_*i*_	Compressor inlet temperature	30°C
*T*_*do*_	Compressor discharge temperature	390°C
*T*_*fo*_	Gas turbine inlet temperature	1085°C
*T*_*eo*_	Gas turbine exhaust temperature	535°C
*P*_*ro*_	Compressor pressure ratio	11.5
*γ*	Ratio of specific heat	1.4
*η*_*c*_	Compressor efficiency	0.85
*η*_*t*_	Turbine efficiency	0.85
R	Speed Regulation	0.04
*T*_*t*_	Temperature control integration rate	0.469
*T*_*c*max_	Temperature control upper limit	1.1
*T*_*c*min_	Temperature control lower limit	0
*F*_*d*max_	Fuel control upper limit	1.5
*F*_*d*min_	Fuel control lower limit	0
*T*_*v*_	Valve positioner time constant	0.05
*T*_*fu*_	Fuel system time constant	0.4
*T*_*w*_	Air control time constant	0.4669
*T*_*cd*_	Compressor volume time constant	0.2
*K*_0_	Gas turbine output coefficient	0.0033
*K*_1_	Steam turbine output coefficient	0.00043
*T*_*g*_	Governor time constant	0.05
*K*_4_	Gain of radiation shield	0.8
*K*_5_	Gain of radiation shield	0.2
*T*_3_	Radiation shield time constant	15
*T*_4_	Thermocouple time constant	2.5
*T*_5_	Temperature control time constant	3.3
*K*_3_	Ratio of fuel adjustment	0.77
*K*_6_	Fuel valve lower limit	0.23
*T*_*m*_	Tube metal heat capacitance time constant of waste heat recovery boiler	5
*T*_*b*_	Boiler storage time constant of waste heat recovery boiler	20
*T*_*i*_	Turbine rotor time constant	18.5
W	Air flow	1.0

- A multi shaft CCGT was considered
- CCGT consisted of two gas turbines and one steam turbine
- Both gas turbines were of 104 MW
- The steam turbine was of 122 MW
- HRSG 330 MW

## Conclusion

A model of CCGT was developed and variation of efficiency by varying various parameters was studied. The results of which can be summarized as follows:-(1) The efficiency of Gas Turbine increases with the maximum cycle temperature ratio. The exhaust temperature of the Gas turbine can be increased up to a limit only due to structural limitations. But inlet Temperature (T1) can be lowered which increases the maximum cycle temperature ratio (T3/T1) which in turn will increase the Gas turbine efficiency (Figure 9).(2) Improving the gas turbine efficiency alone does not necessarily mean the increase in the overall efficiency of the combined cycle (Figure 7). Increasing the gas turbine efficiency would cause lower input steam temperature for steam turbine for given output temperature so the efficiency of the steam turbine would decrease causing the drop in the overall efficiency of the combined cycle (Figure 8).(3) The temperature exhaust of the gas turbine is also an important parameter which has to be maintained as by increasing fuel flow more power output can be obtained but it would cause a rise in the temperature but since the temperature has to be limited below a safe value as an increase in temperature can cause the turbine components to get damaged. The temperature is controlled by more air flow in the turbine (Figure 10, Figure 11 and Figure 12).

(4) With the rise in the ambient temperature of the atmosphere the output of the gas turbine falls and the output of the gas turbine can be increased by reducing the inlet temperature of the compressor by cooling of the air that is being fed to the compressor.

(5) When the fuel input to the turbine is increased, for increasing the output, the air flow has also to be adjusted accordingly to prevent the turbine temperature to go above a reference temperature. There is a linear rise in air flow with the fuel flow when the turbine is being operated near its rated value.

## Appendix

Gas Turbine Model – Frame 6, MS9000 series units, 50 Hz application (rotational speed 3000 rpm).
